# Treatment of Chronic Dysentery

**Published:** 1876-04

**Authors:** 


Treatment of Chronic Dysentery.—Dr. R. E. Thompson writes to the *British Medical Journal:* Four years ago, when I was visiting physician to the Seamen's Hospital, Greenwich, I made several trials of the compound tincture of benzoin in the cases of chronic dysentery that came under my care. I was not aware that the drug had been tried before in this form of disease; but its use was suggested from its effect on mucous membranes generally. The results of my experiments were, that benzoin had no effect in checking the disease or in alleviating the symptoms, and that it was not comparable to small doses of ipecacuanha frequently repeated. Possibly the cases treated by Mr. R. Donaldson differed in some respects from the cases under my care at Greenwich, and I have no intention of questioning the accuracy of his statements. I only wish to state that the medicine has been tried in this country, and that in my hands it has not succeeded. I found it necessary to exercise great caution in admitting the virtues of any drug in this disease, and it is natural in so chronic and wearing a disorder that any remedy is looked to with expectation by the patient, who is always hopeful that some remedy has been found to alleviate his misery; at least, this was my unvaried experience in the numerous trials that I made of various drugs.

The conclusion at which I arrived was, that the disease was best treated by rigidly keeping the patient at rest in bed, in a supine position; by carefully regulating the temperature of the room to about 62 deg. Fahr.; by restricting the diet to few and simple foods, chiefly milk and mutton; and by administering, at frequent short intervals, every three hours, small doses (three to five grains) of the powdered ipecacuanha. If nausea were produced, the dose was diminished or omitted for a time, as I consider that it is very desirable to avoid inducing any disinclination to food. Alcohol in any form very decidedly aggravates the symptoms, and it was always strictly forbidden. These conclusions, perhaps, contain nothing new; but they result from the trial of many drugs, all of which were found far inferior to ipecacuanha.

